# Effects of Transcranial Direct Current Stimulation on GABA and Glx in Children: A pilot study

**DOI:** 10.1371/journal.pone.0222620

**Published:** 2020-01-07

**Authors:** Chidera Nwaroh, Adrianna Giuffre, Lauran Cole, Tiffany Bell, Helen L. Carlson, Frank P. MacMaster, Adam Kirton, Ashley D. Harris

**Affiliations:** 1 Department of Radiology, University of Calgary, Calgary AB, Canada; 2 Alberta Children’s Hospital (ACHRI), Calgary, AB, Canada; 3 Hotchkiss Brain Institute, Calgary, AB, Canada; 4 Child and Adolescent Imaging Research (CAIR) Program, Calgary, AB, Canada; 5 Department of Neuroscience, University of Calgary, Calgary, AB, Canada; 6 Department of Pediatrics, University of Calgary, Calgary, AB, Canada; 7 Department of Psychiatry, University of Calgary, Calgary, AB, Canada; 8 The Mathison Centre for Mental Health Research and Education, University of Calgary, Calgary, AB, Canada; 9 Addictions and Mental Health Strategic Clinical Network, Calgary, AB, Canada; University of Waterloo, CANADA

## Abstract

Transcranial direct current stimulation (tDCS) is a form of non-invasive brain stimulation that safely modulates brain excitability and has therapeutic potential for many conditions. Several studies have shown that anodal tDCS of the primary motor cortex (M1) facilitates motor learning and plasticity, but there is little information about the underlying mechanisms. Using magnetic resonance spectroscopy (MRS), it has been shown that tDCS can affect local levels of γ-aminobutyric acid (GABA) and Glx (a measure of glutamate and glutamine combined) in adults, both of which are known to be associated with skill acquisition and plasticity; however this has yet to be studied in children and adolescents. This study examined GABA and Glx in response to conventional anodal tDCS (a-tDCS) and high definition tDCS (HD-tDCS) targeting the M1 in a pediatric population. Twenty-four typically developing, right-handed children ages 12–18 years participated in five consecutive days of tDCS intervention (sham, a-tDCS or HD-tDCS) targeting the right M1 while training in a fine motor task (Purdue Pegboard Task) with their left hand. Glx and GABA were measured before and after the protocol (at day 5 and 6 weeks) using a PRESS and GABA-edited MEGA-PRESS MRS sequence in the sensorimotor cortices. Glx measured in the left sensorimotor cortex was higher in the HD-tDCS group compared to a-tDCS and sham at 6 weeks (p = 0.001). No changes in GABA were observed in either sensorimotor cortex at any time. These results suggest that neither a-tDCS or HD-tDCS locally affect GABA and Glx in the developing brain and therefore it may demonstrate different responses in adults.

## Introduction

Transcranial direct current stimulation (tDCS) is a form of non-invasive brain stimulation in which a weak electrical current is passed between two electrodes placed on the scalp. Using various tDCS montages, cortical excitability can shift to a state of excitation (anodal tDCS) or inhibitory (cathodal tDCS). Placing the anode electrode over M1 for instance typically increases cortical excitability in M1 [1–3]. Previous research suggests that changes in excitability outlasts the stimulation session by up to 90 minutes [2,4]. The prolonged and promising changes in both cortical excitability and promising changes in behavioral outcomes combined with its simple application and low cost makes tDCS an attractive as a possible therapeutic tool for a range of clinical conditions [5]. For example, tDCS has been suggested to improve symptoms and/or assist in rehabilitation for many neurological disorders with minimal side effects [6], including migraine [7], stroke [8], Parkinson’s disease [9], pain disorders [10] and neurodegenerative disorders [11], as well as psychiatric disorders including depression [12].

High definition tDCS (HD-tDCS) is a newer, more focal form in tDCS that uses arrays of smaller electrodes to improve stimulation localization [13]. Most typically used is the 4 x 1 configuration where a central electrode, which determines montage polarity, is placed over the target cortical region, and four outer electrodes (arranged as a ring), act as the reference electrodes. The radii of the surrounding reference electrodes define the region undergoing modulation [14]. This configuration has been shown to modulate excitability in a smaller, more specific region compared to conventional tDCS [14,15]. In addition to a more focussed current, its effects on patterns of cortical excitability in the M1 outlast those induced by conventional tDCS, as quantified by motor evoked potentials in response to stimulation [16]. Studies support its tolerability in both healthy subjects and patients at intensities up to 2 mA for up to 20 minutes [15–17].

When considering safety of both tDCS and HD-tDCS, in paediatric and adolescent populations, both tDCS and HD-tDCS have been reported as being well tolerated with tingling and itching comparable across all intervention groups including placebo [18]. Applicable adult guidelines are available for tDCS [19,20] and pediatric guidelines to address issues specific to children suggest more moderate dosing is appropriate [21,22]. Our procedures, specifically using 1 mA stimulation, fall well within these guidelines. However, there is known variability between individuals (including differences between sexes) based on cortical folding and skull thickness which has been acknowledged as a limitation of non-invasive brain stimulation [23]. Longitudinal studies investigating the long-term effects of non-invasive brain stimulation are important in building our understanding of tDCS and similar modalities.

Few studies have investigated tDCS in children, despite its potential [20,21,24,25]. tDCS administered in a multiday paradigm to the M1 of healthy children while performing a motor task demonstrated greater increases in motor skill compared to sham and improvements are retained 6 weeks later [18,26]. These findings suggest the potential utility of tDCS as a therapeutic tool in children with motor impairments but the biological mechanisms behind these effects remain unknown [27].

Adult studies using magnetic resonance spectroscopy (MRS) to measure regional brain metabolites typically show a decrease in GABA [4,28,29] and an increase in Glx (glutamate and glutamine in combination) [4,29,30] in the sensorimotor cortex following M1 anodal stimulation. Both GABA, a major inhibitory neurotransmitter, and glutamate, a major excitatory neurotransmitter, are mediators in long-term potentiation [31,32] and have been associated with behavioral changes following anodal tDCS, quantified as changes in task performance [4,28,33]. However, it is unknown if these finding translate to a pediatric population and how long these changes in metabolites persist.

Point RESolved Spectroscopy (PRESS) at 3T measures glutamate, N-Acetyl Aspartate (NAA), creatine (Cr) and choline (Cho). Glutamate it is often reported as Glx, representing the combination of glutamate and glutamine as their spectra are highly overlapped, making it difficult to reliably resolve these two signals. GABA, on the other hand, is at low concentration and its signal is overlapped by more abundant metabolites and therefore requires editing for accurate measurement [34]. GABA-edited MEGA-PRESS, selectively manipulates the GABA signal at 3 ppm by applying an editing pulse to the coupled GABA signal at 1.9 ppm in half of the averages (ON), which are interleaved with averages in which the editing pulse is applied elsewhere not coupled to GABA (OFF). The difference spectrum is acquired by subtracting the ON from the OFF, which removes all peaks not affected by the 1.9 ppm editing pulse (specifically the 3 ppm creatine peak), revealing the GABA signal at 3 ppm. While information on Glx, NAA, Cr and Cho are available in the GABA-edited MEGA-PRESS data, there is not much data available validating these measures. The editing pulse in GABA-edited MEGA-PRESS does not directly target the peak at 2.1 ppm so the co-edited glutamate and glutamine peaks are only partially refocused resulting in a fraction of the possible signal present in the difference spectra [34,35]. The OFF sub-spectrum is an alternative but the longer TE in the MEGA-PRESS sequence results in greater signal decay compared to the short-echo PRESS.

In this study, GABA-edited MEGA-PRESS and PRESS MRS sequences were used to investigate changes in GABA and Glx in response to anodal tDCS (a-tDCS) and anodal high definition tDCS (HD-tDCS) in a pediatric population with the tDCS anode targeting the motor cortex and participants performing a simple motor task to assess learning. By observing metabolite changes in the targeted right sensorimotor cortex and the contralateral left sensorimotor cortex, we aimed to gain insight into the metabolite changes induced by tDCS both after stimulation has concluded and at 6 weeks follow up, with the overall goal of better understanding the mechanism by which tDCS modulates motor learning in the developing brain. Based on the adult literature, we expected GABA to decrease following tDCS and at 6-weeks follow up we expect metabolites to return towards baseline with similar results observed for both anodal and high definition tDCS groups.

## Materials and methods

This study was a component of the Accelerated Motor Learning in Pediatrics (AMPED) study, a randomized, double-blind, single-center, sham-controlled intervention trial registered at clinicaltrials.gov (NCT03193580) with ethics approval from the University of Calgary Research Ethics Board (REB16-2474) to be performed at the Alberta Children’s Hospital. The results described here are from the registered secondary outcome measure #1. Upon enrolment, participants and guardians provided written, informed consent or assent and were screened to ensure they met safety criteria for non-invasive brain stimulation and MRI scanning. Participants were blinded to the experimental group to which they were assigned and only the investigator administering stimulation was aware of the group until all data was collected. Experimental groups were assigned using an automated number generator. Group assignment was only revealed to those assessing outcomes for data analysis after the study was completed. Additional details regarding the parent study design, recruitment and primary motor learning outcomes can be found in Cole and Giuffre et al [18].

### Experimental design

**Fig 1:**
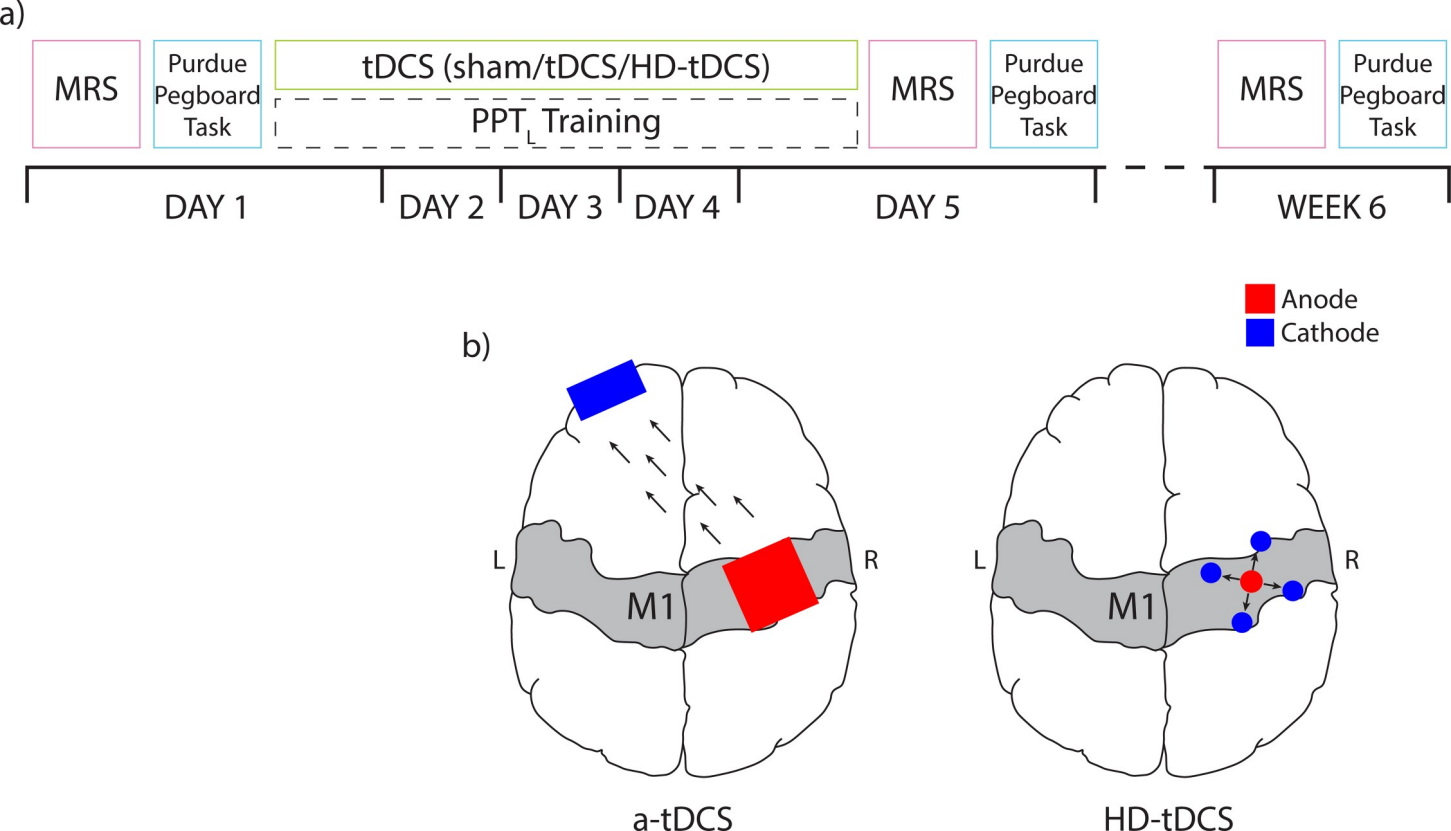
Layout of experimental procedure. a) On Day 1, spectroscopy measurements were collected followed by the Purdue Pegboard Task. Participants then underwent five consecutive days of right M1 targeted anodal tDCS paired with left hand motor training. Participants repeated Day 1 assessments after intervention on Day 5 and at 6 week follow up. b) Anodal tDCS electrode montages shown for a-tDCS (left) and HD-tDCS (right) intervention groups where the anode is red, the cathode is blue and current flow is illustrated with black arrows. MRS, magnetic resonance spectroscopy; PPT, Purdue pegboard task, tDCS, transcranial direct current stimulation; HD-tDCS, high definition tDCS.

Twenty-four typically developing right-handed participants ages 12 to 18 were recruited through the Healthy Infants and Children Clinical Research (HICCUP) Database. The Edinburgh Handedness Inventory, a measurement used to asses and individuals hand dominance in everyday activities [36], was used to confirm right hand dominance (a laterality index ≥ -28). The Edinburgh Handedness Inventory ranges from -100 for completely left hand dominant to +100 for completely right hand dominant, thus a threshold of ≥ -28 ensures right-handedness. We chose to recruit right-handed individuals and target the right motor cortex and measure motor performance in the left, non-dominant hand. This ensures that plasticity was being modulated in the non-dominant cortex. Participants were excluded for MRI contraindications, neuropsychiatric or developmental disorder diagnoses, medications or pregnancy. All participants received a baseline MR scan and motor assessments prior to tDCS. Participants were then computer randomized to a single tDCS condition (n = 8 for each intervention group) with the anode targeting right M1: a-tDCS (1mA conventional anodal tDCS), HD-tDCS (1mA high definition anodal tDCS) and sham tDCS. Participants took part in a 5-day protocol in which they received stimulation each day whilst training in the Purdue Pegboard Task (PPT) using their non-dominant left hand. Participants repeated the PPT task 3 times during the stimulation period and the average score was taken. Immediately following stimulation, participants completed the PPT for a final time and PPT task performance was quantified based on this final PPT performance. After stimulation had concluded on Day 5, they received a post-simulation MR scan and completed all motor assessments. Participants returned 6 weeks (± 1 week) later for a follow-up MR scan and motor assessments. The experimental design for this study is shown in Fig 1A.

### Transcranial direct current stimulation

Participants received 20 minutes of 1mA anodal tDCS in a montage dependent on the assigned stimulation condition. tDCS was administered using a conventional 1 x 1 tDCS or a 4 x 1 HD-tDCS system (Soterix Medical Inc., New York, USA) (Fig 1B). For participants in the a-tDCS or sham group, two 25 cm^2^ saline-soaked sponge electrodes were held on the scalp using a light plastic headband (SNAPstrap, Soterix Medical Inc., New York, USA). The active (anodal) electrode was centred on the right M1 (identified using robotic single pulse transcranial magnetic stimulation; TMS) and the cathodal electrode was placed on the contralateral supraorbital notch, an inert location. The electrodes were connected to a 1 x 1 DC SMARTscan Stimulator (Soterix). This montage demonstrated in Fig 1B has been used extensively in tDCS studies for motor training in the non-dominant left hand [4,18,25,28,37,38].

For the HD-tDCS group, a 10:20 EEG cap was used to center the anodal electrode on the right M1, after identifying the location with single pulse TMS as above. The four cathodes were placed ~5 cm away in a 4 x 1 configuration (Fig 1B) using a 4 x 1 HD-tDCS Adaptor and a SMARTscan Stimulator (Soterix) as described previously [15,39,40]. 1mA is the standard for anodal tDCS in children at our institution and has been shown to elicit improvements in motor learning and chances in spectroscopic biomarkers [18,26,33]. While many use 2 mA stimulation parameters, 1 mA is also commonly used [4,24,38,40,41]. Additionally, current modeling investigations report variations in electric fields between adults and adolescents associated with developmental differences in skull thickness and grey and white matter, suggesting 1mA is more appropriate for children [42].

For the active stimulation conditions (a-tDCS and HD-tDCS), current was ramped up to 1 mA over 30 seconds and remained at 1mA for 20 minutes. The current was then ramped back down to 0 mA over 30 seconds. For the sham stimulation condition, current was ramped up to 1 mA over 30 seconds and then immediately ramped back down to 0 mA over 30 seconds. After 20 minutes, current was ramped up to 1 mA and then back down to 0 mA over 30 seconds. This procedure is used to mimic the sensations associated with active stimulation and has been previously validated [43]. During the 20 mins of stimulation (or sham) participants performed the Purdue Pegboard Task with their left hand (PPT_L_) three every 5 minutes.

The operator applying the stimulation was the only person aware of the type of stimulation that was being applied. Participants were naive to tDCS and unaware that a sham or HD-tDCS intervention group existed, though we acknowledge that participants may be able to differentiate HD-tDCS due to the difference in electrode configuration. As reported Cole and Giuffre et al., participants were not able to successfully guess their treatment group supporting effective blinding [18].

### Motor assessments

The motor assessment was the Purdue Pegboard Task (PPT) [44]. This test uses a rectangular board with two sets of 25 holes running vertically down the board and four concave cups at the top of the board that contain small metal pegs. Subjects are asked to remove pegs from the cups and place them in the holes one-at-a-time, as quickly as possible. This task challenges hand dexterity and coordination. A score is given as the number of pegs successfully placed in the holes in 30 seconds with the left hand (PPT_L_). Secondary assessments were the performance of this task with the right hand (PPT_R_) or bimanually (PPT_LR_). Changes in score is reported as ΔPPT.

### MRS acquisition

**Fig 2:**
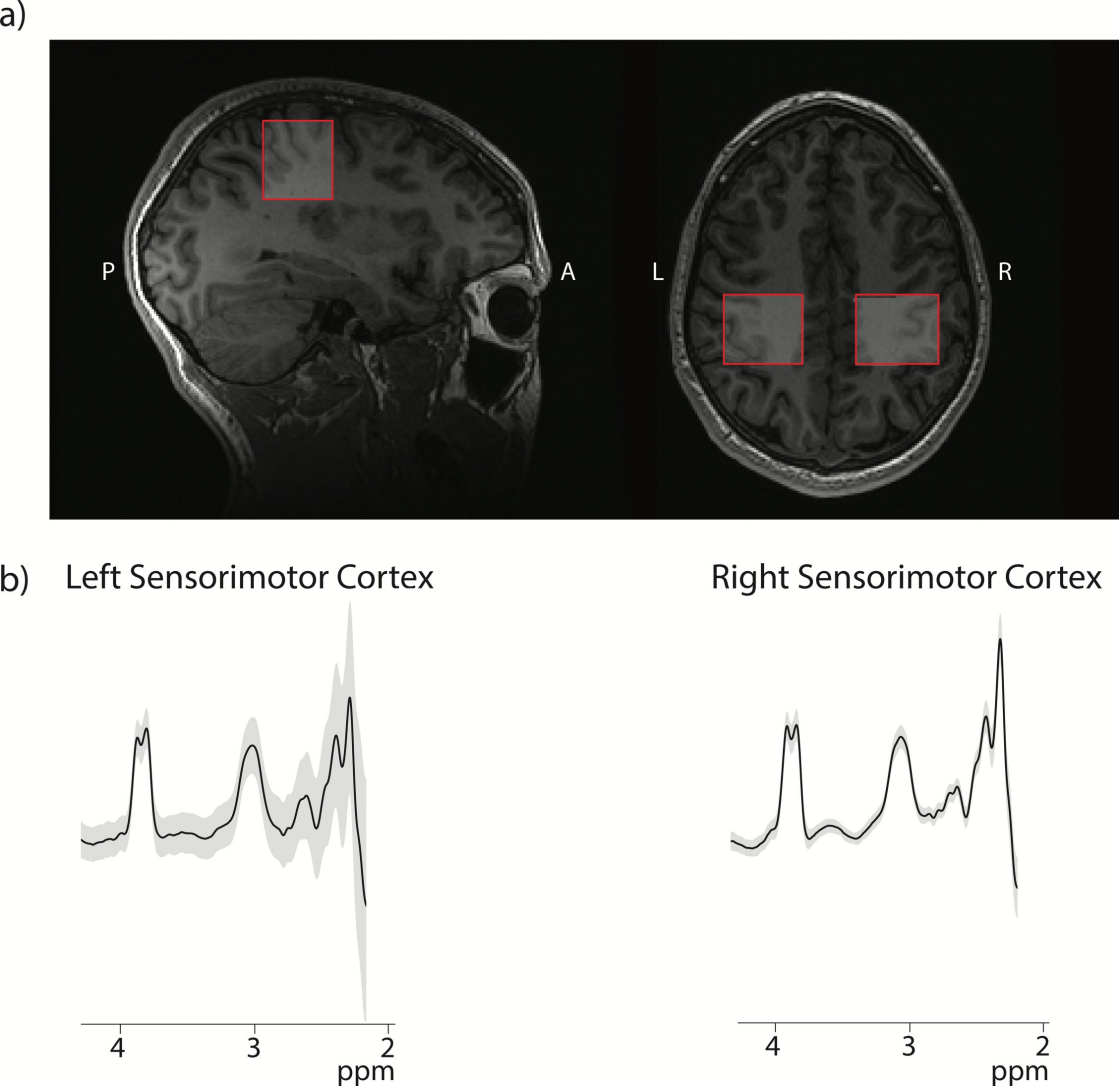
Voxel placement and data quality. Example of voxel placement in the sensorimotor cortex on a participant T1-weighted image. b) GABA-edited MEGA-PRESS spectra acquired in each location. The black line depicts the average fit line and the grey area shows ±1 standard deviation in the right and left sensorimotor cortex.

Spectroscopy data was collected before the tDCS intervention (baseline), after 5 days of tDCS paired with motor training, and at 6-weeks after tDCS in all 24 subjects on a 3T GE MRI scanner equipped with a 32-channel head coil. Axial T1-weighted fast spoiled gradient recalled echo (FSPGR) brain volume images (BRAVO) were acquired (TR = 7.4 ms, TE = 2.8 ms with 1 mm^3^ voxels) for voxel placement and tissue segmentation. Metabolites were measured in 30×30×30 mm^3^ voxels located on the right and left sensorimotor cortices. The sensorimotor cortex was identified by Yousry’s hand-knob [45] and the voxel was rotated to align with the cortical surface (Fig 2). GABA data were acquired using a GABA-edited MEGA-PRESS sequence with the following parameters: TR/TE = 1800/68 ms, 256 averages; 14 ms editing pulses applied at 1.9 ppm and 7.46 ppm alternating every two averages, and 16 unsuppressed water scans. A PRESS sequence was used to acquire MRS data to quantify Glx, Cr, Cho and NAA with the following parameters: TR/TE = 1800/35 ms, 64 averages and 8 unsuppressed water scans. In order to perform symmetrical assessment of the left and right sensorimotor cortices, the water-fat shift directions were mirrored for the sensorimotor voxels for both the GABA-edited MEGA-PRESS and the PRESS acquisitions.

### MRS data analysis

GABA data were analyzed using GANNET 3.0 [46] software in MATLAB R2014a (The Mathworks, Natick, MA, USA), including retrospective frequency and phase correction and correction for voxel tissue content for each voxel, including the assumption that the concentration of GABA in grey matter is twice that of white matter (i.e., α = 0.5 as per literature) [47]. This correction accounts for individual voxel composition as well as accounting for differences in GABA concentrations between cerebrospinal fluid, white matter and grey matter and reduces inter-subject variance in concentration that is driven by voxel-tissue compositions [35,48]. In this experiment, after tissue correction, we normalized the sensorimotor voxel data to represent voxels that were composed of 40% grey matter and 60% white matter such that the right and left data could be directly compared [49]. PRESS data was corrected for frequency and phase drift using the FID-A toolkit [50] and then analyzed using LCModel [51] with basis sets developed from LCModel. Metabolite levels from LCModel were tissue-corrected using the Gasparovic approach [48] using tissue segmentation from GANNET and the CSF voxel fraction, accounting for the negligible metabolites present in CSF. As a confirmatory analysis, metabolite levels referenced to creatine were also examined.

### Statistical analysis

All statistical analyses were performed using SPSS Statistics 25 (IBM, Armonk, NY, USA). Demographic data of the three groups (a-tDCS, HD-tDCS and sham) were compared with an ANOVA model and Chi-squared for sex data. Changes in GABA and Glx between tDCS conditions and over time were assessed using a linear mixed model analysis with fixed effects for intervention and experimental day, the interaction of intervention and experimental day, and covariates for age and sex for each voxel. Post-hoc pair-wise analyses with Bonferroni correction for multiple comparisons were performed to specifically examine effects of intervention and experimental day.

Partial correlations controlling for intervention were used to examine the relationship between changes in metabolites and changes in motor assessment performance before and after stimulation, and 6 weeks after stimulation had concluded. Initially these correlations were pooled across all groups and follow-up analyses were performed in each group as appropriate.

## Results

### Population characteristics

Twenty-four typically developing children (mean 15.5 ± 1.7 years, 13 females and 11 males) completed all phases of the study with no dropouts. No adverse events were reported during this investigation. Due to technical difficulties, one participant did not have GABA or Glx data available in both sensorimotor cortices in the post intervention timepoint. Population demographics are shown in Table 1. Age was not controlled within each experimental group however age and laterality index did not differ significantly between groups. While there is a noted difference between gender between group, our statistical model controlled for gender. However, we acknowledge that the variation in gender between groups has the potential to affect results.

**Table 1:** Mean participant demographics ± 1 standard deviation for all stimulation intervention groups. No significant difference between groups was identified.

	SHAM (±SD)	a-tDCS (± SD)	HD-tDCS (± SD)	MEAN (± SD)
**AGE**	15.81 (±1.3)	15.94 (± 1.5)	14.77 (± 2.0)	15.51 (± 1.7)
**LATERALITY INDEX**	81.9 (± 22.8)	82.5 (± 13.1)	81.3 (± 14.7)	81.9 (± 16.6)
**SEX (M:F)**	2:6	5:3	4:4	11:13

### Data quality

The GABA-edited MEGA-PRESS spectra from the right and left sensorimotor cortices from all time points are show in Fig 2B; the grey area shows a single standard deviation range across all data and the black line is the average of all data. All data, both GABA-edited MEGA-PRESS and PRESS, were assessed for quality by visual inspection as well as a CRLB threshold of 20%. One PRESS dataset was excluded due to poor data quality, the remaining spectra were of high quality with a mean SNR of 41.4±6.3, all FWHM water <15 Hz, mean FWHM water 6.01±1.92 Hz. MEGA-PRESS GABA data was also of high quality across all data sets: all fit errors < 10%, mean fit error 4.59±1.21, all FWHM Cr <10%, mean FWHM Cr: 9.57±0.92 Hz. Generally, spectra with fit errors below 12% are deemed to be of sufficient quality [46].

### Metabolite group changes

**Fig 3:**
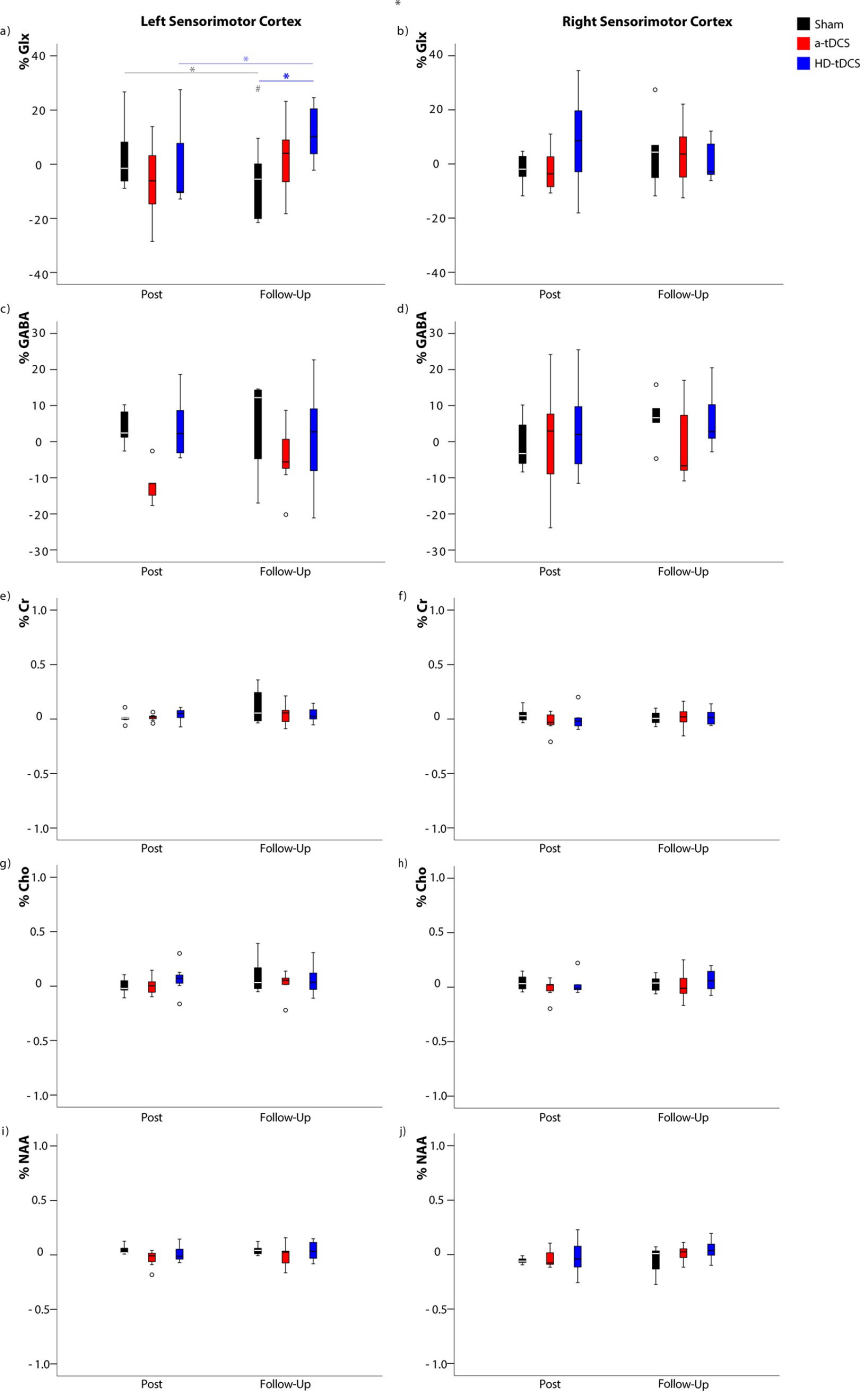
Metabolite changes over time. Pairwise comparison for changes in metabolite levels for all intervention groups (sham in black, tDCS in red and HD-tDCS in blue) over the duration of the experiment given as a percentage change from baseline values (mean ± 1 SD). * p < 0.05, those in bold withstand Bonferroni correction for multiple comparisons while those that are transparent lose significance following multiple comparisons correction. # p < 0.05 when compared to baseline. Cr: Creatine, Cho: Choline, NAA: N-Actylaspartic Acid.

Linear mixed model analyses showed a significant fixed effect of tDCS intervention over time on Glx levels in the left sensorimotor cortex (effect size estimate = 10.38, df = 61.00, p = 0.010). Post-hoc Bonferroni corrected pairwise analyses showed at the 6 week follow up, Glx was significantly higher in the HD-tDCS group compared to the sham group (p = 0.001; Fig 3). In the HD-tDCS group, Glx in the left sensorimotor cortex increased between post-intervention and the 6 week follow up time points (p = 0.042), however, this did not withstand correction for multiple comparisons (Fig 3). No significant fixed effect of tDCS intervention over time for Glx was detected in the right sensorimotor cortex (effect size estimate = 2.31, df = 57.04, p = 0.221). No significant fixed effect was observed in the left sensorimotor cortex for GABA (effect size estimate = 3.91, df = 53.71, p = 0.248), creatine (effect size estimate = 0.0172, df = 54.20, p = 0.425), choline (effect size estimate = 0.0631, df = 51.47, p = 0.572) or NAA (effect size estimate = 0.0377, df = 49.81, p = 0.177). The same was true in the right sensorimotor cortex for GABA (effect size estimate = 4.71, df = 55.95, p = 0.724), creatine (effect size estimate = 0.0389, df = 61.30, p = 0.246), choline (effect size estimate = 0.0556, df = 48.77, p = 0.483) or NAA (effect size estimate = 0.0419, df = 52.73, p = 0.458). Metabolite data referenced to creatine showed the same results. No significant metabolite change difference were detected between that a-tDCS and sham groups in both left and right sensorimotor cortices.

### Relationship between metabolite changes and motor performance

**Fig 4:**
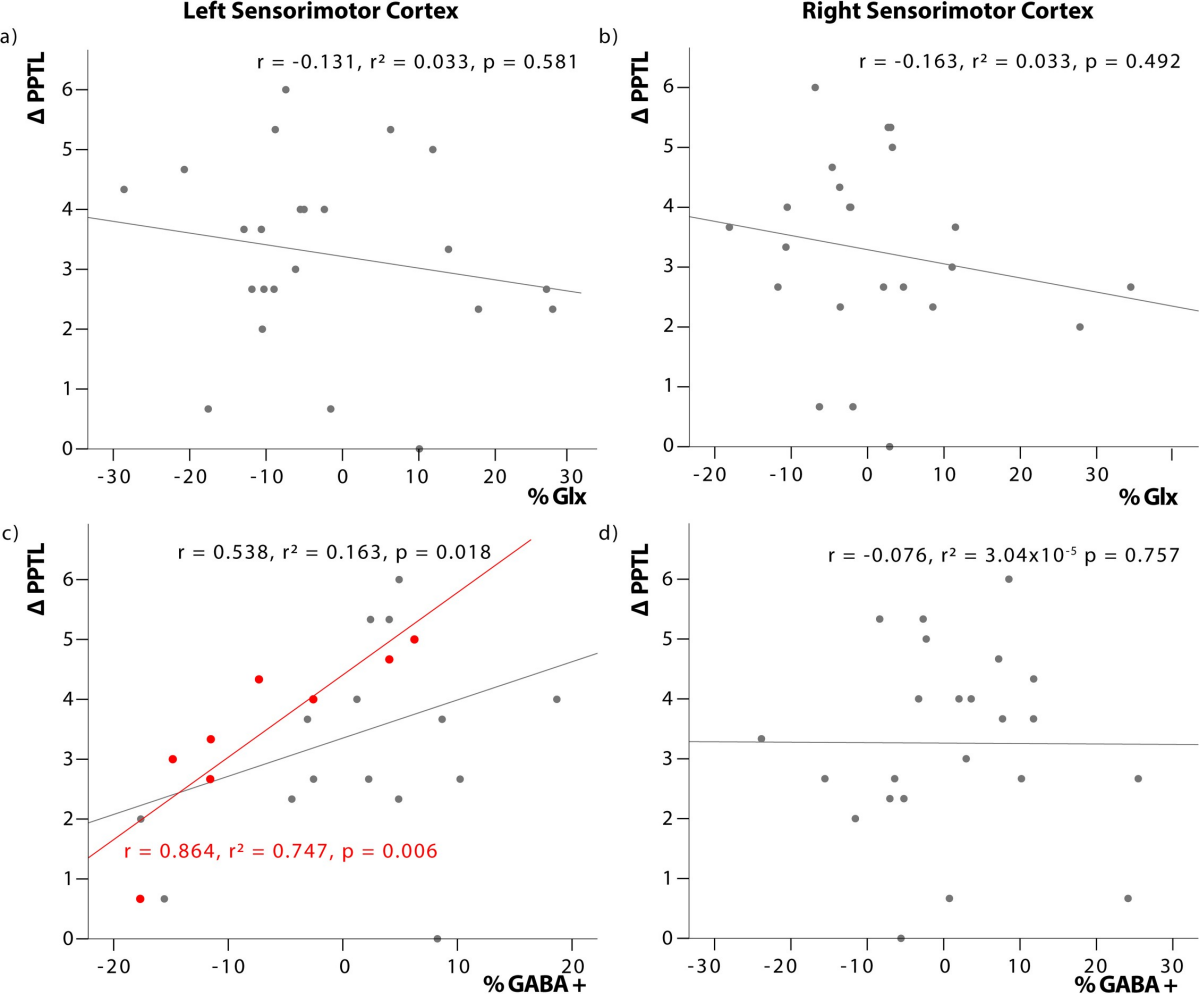
Relationship between changes in metabolite concentration and motor performance. Correlationn between change in metabolite concentration (% Glx and %GABA) and change in Purdue Pegboard Task post intervention (ΔPPT_L_) controlling for intervention group and age. Left sensorimotor cortex GABA is significantly correlated with PPT_L_ for the pooled intervention groups (grey line). This relationship is also observed in the anodal tDCS intervention group (red).

Partial correlation analysis comparing changes in GABA and Glx, pooled across the three intervention groups, showed a significant positive relationship between the change in left sensorimotor GABA (%GABA) and change in PPT_L_ score (ΔPPT_L_) (r = 0.538, p = 0.018; Fig 4C), participants with a greater positive change in GABA showed a greater improvement in PPT. Post-hoc assessments by intervention groups showed this relationship was maintained in the anodal tDCS group only (r = 0.864, p = 0.006; Fig 4C).

No significant relationship was observed between ΔPPT_L_ and changes in GABA in the right sensorimotor cortex (r = -0.076, p = 0.757; Fig 4D). Additionally, no significant relationship was seen between changes in PPT score and changes in Glx in the left (Fig 4A) (p = 0.581) or right (Fig 4B) sensorimotor cortex (p = 0.492).

## Discussion

Several adult studies have shown that single [52,53] or multiple session [37,54] tDCS paired with training in a motor task is associated with improvements in said task and improvements in performance are greater than motor training alone (i.e., sham-tDCS). The same is observed in pediatric studies [18,26], however results may differ slightly in terms of the phase of learning affected by stimulation. Results in children suggest that tDCS facilitates online learning [26] while in adults evidence suggests tDCS enhances learning primarily through offline effects [37]. GABA and glutamate are involved in learning [28,32,55] and have both been observed to change in response to anodal tDCS in adults [4,28–30,55,56]. This study examined changes in GABA and Glx in response to right M1 anodal tDCS and HD-tDCS in a pediatric population. Metabolites were measured at baseline, after a 5-day tDCS and motor learning intervention (post-intervention) and at 6 weeks follow-up.

To our knowledge, this is the first investigation of metabolite changes in response to tDCS in a typically developing pediatric population. Additionally, this is the first-time metabolites have been measured in a control population after a multiday protocol with a follow-up assessment. Previous studies in adults have illustrated that GABA decreases [38,55] and glutamate increases [57], with skill acquisition and improved function in the region responsible for the skill execution, the M1. It has been suggested that tDCS facilitates changes in GABA and glutamate to augment learning. Studies conducted in adults have shown anodal tDCS increases sensorimotor glutamate [4,29,30] and decreases GABA [4,28,29,58]; however, others have failed to replicate these findings. Similarly, we did not see decreased GABA and increased Glx at the site of stimulation, though we did see contralateral changes. Our results potentially indicate the developing brain responds differently to tDCS compared to the adult brain.

### Post-intervention changes in GABA and Glx

Following five days of tDCS and motor training there were no significant changes in metabolite levels in either the right or left sensorimotor cortex, though trends toward decreased left sensorimotor GABA (contralateral to the tDCS target) in the a-tDCS group were seen. Adult literature using healthy controls suggests acute decrease in GABA local to the tDCS target [4,28,29,58]. Similarly, participants with a neurodegenerative condition who followed a protocol of 15 a-tDCS sessions also showed decreased GABA in the tissue targeted with a-tDCS [11]. Given the contrast of our results and those in the literature, we suggest that the pediatric brain responds differently to tDCS. However, due to our limited sample size this interpretation is not concrete.

In healthy adults, GABA and glutamate in the motor cortex work together to maintain an excitation-inhibition balance that is crucial for plasticity [59]. It has been suggested that this balance of GABA and glutamate can be shifted to a relative optimum level that is thought to mediate behavioral outcomes [60]. It is possible that in the developing brain, this excitation/inhibition balance is more dynamic while in the adult brain it is relatively static. When an external stimulus is introduced, like tDCS or a foreign motor task, the adult brain shows a shift to facilitate plasticity while the pediatric brain was already in its “plastic state”. There is also evidence describing the pediatric brain as being hyperexcitable with dynamic changes in glutamatergic excitatory and GABAergic inhibitory mechanisms during development [24], and therefore less dynamic range to reduce GABA compared to the adult brain where increased GABAergic inhibition is necessary to refine already acquired skills. However, there is literature demonstrating widespread grey and white matter maturation throughout adolescence into adulthood that is thought to be associated with neural development [61] so it is likely that there is no clear transition point where the pediatric brain loses its plasticity and settles into a more static adult state.

Secondly, transcallosal inhibitory processes [62] may have a more pronounced effect in the pediatric brain. Here we show trends towards decreased GABA in the left sensorimotor cortex, contralateral to the site of stimulation, as opposed to changes in the site of stimulation (right cortex). This suggests lateralization of motor learning in the left dominant cortex as previously described by Schambra et al [63]. The impact of transcallosal inhibition is also seen in pediatric studies applying tDCS contralateral to stroke lesions in an effort to augment motor learning of the affected hemisphere [42,64]. According to pediatric models of anodal tDCS, the current appears to travel through the motor fibers of the corpus callosum into the contralateral hemisphere [42]. However, the same mechanism is not expected to be true for HD-tDCS which has a more focal current.

Finally, as mentioned above, tDCS may act on different phases of learning in children compared to adults, therefore the paradigm in which we expect GABA and glutamate changes to appear shortly after stimulation is not the appropriate time window to detect changes. Similarly, it is possible that the metabolic response to stimulation changes with applications over consecutive days. In this study, we suspect participants may have transitioned into a phase of learning that requires less plasticity and the cortex is no longer responding to tDCS with the predicted GABA and Glx changes at five days when our measures were taken. Adult literature suggests the changes in GABA and glutamate measured by MRS in response to learning vary with time [55,65] and it is possible that a ceiling of PPT skill, and also of metabolite change, was reached before our MRS measurements were taken.

Although no significant changes in GABA concentration were detected between MRS measurements, this does not conclusively rule out GABAergic changes in response to motor learning. It is possible that subtle biphasic changes in GABA are taking place during motor learning that we are unable to detect. While this cannot be confirmed in our investigation, there is literature suggesting changes in GABA concentration are time sensitive with fluctuation in GABA concentration occurring in the 90 minute window following stimulation [4,55,65]. The time sensitivity of metabolite measurements is further supported by seemingly discrepant findings in the literature in which GABA and Glx changes are not seen during tDCS [66–68].

Changes in Glx in response to stimulation in the literature are inconsistent. Clark et al. reports Glx increases after anodal tDCS and suggest that tDCS may involve the NMDA pathway [30]. Stagg et al. also reports changes in Glx in response to cathodal tDCS, but not anodal tDCS [4]. They propose MRS measures of Glx lack sensitivity to consistently detect Glx changes following tDCS [4,28]. Several other studies report an absence of significant changes in Glx in response to a-tDCS at the site of stimulation with little speculation as to why [4,29,66,67,69]. It may be that tDCS influences Glx in a complex way that is network dependent. The results of the current study shows Glx changes in the sensorimotor cortex contralateral to the site of stimulation could be consistent with observations of others who have also seen changes in Glx remote from the site of the tDCS [66,70].

### 6 week follow up in GABA and Glx

At 6 weeks follow up, it was expected that metabolites would return to baseline to maintain homeostatic balance in the brain after the initial phases of skill acquisition had concluded, while retaining motor skill improvements. However, we observed a significant increase in the left sensorimotor Glx at 6 weeks follow up in the HD-tDCS group compared to the sham group (p = 0.001) and compared to HD-tDCS baseline level. We also see a trend of increased Glx in the HD-tDCS group between post intervention and 6-week follow up in the left sensorimotor cortex [18]. These observed trends must be interpreted cautiously as the sample size is small. If this result is replicated, it suggests that in the hemisphere contralateral to stimulation, HD-tDCS has a longer-term modulation of glutamatergic pathways. When examined in conjunction with the secondary motor data collected, the change in left sensorimotor Glx in the HD-tDCS group is accompanied by an improvement in the right hand PPT at 6 weeks follow up. A potential explanation is motor overflow, a phenomena that typically disappears in late childhood and describes unintentional movement that mirror voluntary movements typically in homologous muscle on the opposite side of the body [71]. Similarly, the decrease in GABA in the left sensorimotor cortex in the a-tDCS group persisted. Persistent decreases in GABA several weeks after tDCS intervention have been seen in primary progressive aphasia [11]; though those changes were seen at the site of stimulation. Several studies have shown improvement in motor learning in the contralateral hand following tDCS of either the right or left M1 [52,72,73]. The “callosal access” hypothesis suggests that performance can be facilitated in the untrained limb due to motor engrams developed in the dominant hemisphere. These engrams underlie performance of the trained hand located in homologous regions that the opposite motor cortex can access via the corpus callosum [64,74,75].

### Relationship between changes in metabolites and changes in motor performance

We found a significant, positive relationship between change in left sensorimotor GABA (cortex contralateral to stimulation) and improvement in the task performance by the left hand post tDCS intervention and training, further supporting the above mentioned callosal hypothesis. Those participants who experience a greater positive change in GABA concentration in the hemisphere contralateral to stimulation (left motor cortex) present a greater improvement in PPT score over the 5-day stimulation and training period. This relationship is specifically seen in the a-tDCS group only, suggesting that anodal stimulation induces a contralateral inhibition that does not occur with HD-tDCS or in normal (sham group) learning, driving an enhanced improvement in PPT score.

No relationship between changes in Glx and task performance post-intervention nor between GABA or Glx and change in PPT score 6 weeks after stimulation and training was observed. These results are in accordance with adult studies that report no significant relationship between change in motor skill and concentration of Glx in the motor cortex contralateral to the hand executing the task [38]. However, adult studies have reported a relationship between task improvement and GABA changes in the tDCS targeted cortex (i.e. right sensorimotor GABA changes and left hand training and task performance) [28,38]. This dissimilarity suggests that neurochemistry in the pediatric and adult brain respond in different ways during motor learning, warranting further investigation.

## Conclusions

Non-invasive brain stimulation is an expanding area of research with investigations into the use of modalities similar to tDCS being investigated as a therapy for a range of disorders including migraine, pain and stroke [6–8,10,12,20,41]. While these studies have suggested that non-invasive brain stimulation can improve outcomes, the underlying physiological changes behind these responses are not well understood, particularly in the developing brain. Using a control population, this study aimed to better understand the metabolite changes induced by M1 anodal tDCS in conjunction with a motor training paradigm in the developing brain.

We investigated changes, in GABA and Glx concentrations following 5 consecutive days of tDCS comparing conventional anodal tDCS, HD-tDCS and sham. Unexpectedly, transcranial direct current stimulation (tDCS) did not produces localized and specific alterations in neurochemistry at the site of stimulation post 5-day tDCS intervention or 6 weeks after the intervention. It is possible that changes in metabolites occur immediately after stimulation and learning and this effect is diminished over the 5 days stimulation as skill level improves. However, we suggest the pediatric brain responds differently to tDCS compared to adults. In particular, we suggest contralateral modulation of learning and metabolites has a greater role in the pediatric brain, highlighting the need for further study of the effects of non-invasive stimulation on the pediatric brain specifically. Furthermore, we also show the response to HD-tDCS is different compared to a-tDCS based on the observation of increased Glx in the left sensorimotor cortex 6 weeks after stimulation specifically in response to HD-tDCS. Further investigation into the effects of HD-tDCS is needed to determine its efficacy on motor learning.

## Supporting information

S1 Table Tissue corrected metabolite concentrations for each intervention group ± 1 standard deviation. Mean metabolite concentration for GABA and Glx in iu at three experimental time points for each intervention group; anodal tDCS (a-tDCS), anodal high definition tDCS (HD-tDCS) and sham.
(DOCX)
