# Supplementary material for: Effects of Transcranial Direct Current Stimulation on GABA and Glx in Children: A pilot study
Source: PLoS One. 2020 Jan 7;15(1):e0222620. doi: 10.1371/journal.pone.0222620 (PMC6946135; doi:10.1371/journal.pone.0222620)
Supplement: S1 Table — Mean metabolite concentration for GABA and Glx in iu at three experimental time points for each intervention group; anodal tDCS (a-tDCS), anodal high definition tDCS (HD-tDCS) and sham. (DOCX) [file pone.0222620.s001.docx]

|  |  | a-Sham  ($\text{±}$ SD) | tDCS  ($\text{±}$ SD) | HD-tDCS  ($\text{±}$ SD) | Mean  ($\text{±}$ SD) |
| --- | --- | --- | --- | --- | --- |
| Baseline | GABA |  |  |  |  |
|  | RSM | 1.83  ($\text{±}$ 0.16) | 1.96  ($\text{±}$ 0.21) | 1.80  ($\text{±}$ 0.21) | 1.86  ($\text{±}$ 0.20) |
|  | LSM | 1.87  ($\text{±}$ 0.15) | 2.09  ($\text{±}$ 0.09) | 1.96  ($\text{±}$ 0.21) | 1.97  ($\text{±}$ 0.16) |
|  | Thalamus | 2.77  ($\text{±}$ 0.31) | 2.63  ($\text{±}$ 0.45) | 2.91  ($\text{±}$ 0.35) | 2.77  ($\text{±}$ 0.38) |
|  | Glx |  |  |  |  |
|  | RSM | 11.82  ($\text{±}$ 1.08) | 11.79  ($\text{±}$ 0.94) | 11.36  ($\text{±}$ 1.30) | 11.66  ($\text{±}$ 1.09) |
|  | LSM | 11.25  ($\text{±}$ 0.83) | 12.45  ($\text{±}$ 2.37) | 12.36  ($\text{±}$ 2.01) | 12.02  ($\text{±}$ 1.86) |
|  | Thalamus | 11.45  ($\text{±}$ 0.95) | 12.66  ($\text{±}$ 1.27) | 13.20  ($\text{±}$ 1.94) | 12.52  ($\text{±}$ 1.59) |
| Post Intervention | GABA |  |  |  |  |
|  | RSM | 1.84  ($\text{±}$0.13) | 1.95  ($\text{±}$0.20) | 1.83  ($\text{±}$0.15) | 1.88  ($\text{±}$0.17) |
|  | LSM | 1.88  ($\text{±}$0.17) | 1.94  ($\text{±}$0.16) | 2.06  ($\text{±}$0.09) | 1.96  ($\text{±}$0.16) |
|  | Thalamus | 2.92  ($\text{±}$0.36) | 2.85  ($\text{±}$0.36) | 2.70  ($\text{±}$0.32) | 2.82  ($\text{±}$0.33) |
|  | Glx |  |  |  |  |
|  | RSM | 11.41  ($\text{±}$0.77) | 11.47  ($\text{±}$0.87) | 12.08  ($\text{±}$1.18) | 11.67  ($\text{±}$0.97) |
|  | LSM | 11.52  ($\text{±}$1.10) | 11.18  ($\text{±}$1.13) | 11.97  ($\text{±}$0.79) | 11.55  ($\text{±}$1.03) |
|  | Thalamus | 13.24  ($\text{±}$1.99) | 12.59  ($\text{±}$2.00) | 14.08  ($\text{±}$2.89) | 13.03  ($\text{±}$2.31) |
| 6 Week Follow Up | GABA |  |  |  |  |
|  | RSM | 1.90  ($\text{±}$0.10) | 1.87  ($\text{±}$0.11) | 1.80  ($\text{±}$0.10) | 1.85  ($\text{±}$0.12) |
|  | LSM | 2.01  ($\text{±}$0.30) | 2.00  ($\text{±}$0.14) | 2.02  ($\text{±}$0.22) | 2.01  ($\text{±}$0.22) |
|  | Thalamus | 2.96  ($\text{±}$0.27) | 2.75  ($\text{±}$0.18) | 2.90  ($\text{±}$0.18) | 2.88  ($\text{±}$0.22) |
|  | Glx |  |  |  |  |
|  | RSM | 11.93  ($\text{±}$0.71) | 11.97  ($\text{±}$1.01) | 11.96  ($\text{±}$0.96) | 11.96  ($\text{±}$0.88) |
|  | LSM | 11.57  ($\text{±}$0.68) | 12.14  ($\text{±}$1.28) | 12.63  ($\text{±}$1.17) | 12.11  ($\text{±}$1.12) |
|  | Thalamus | 12.77  ($\text{±}$1.91) | 14.39  ($\text{±}$3.04) | 13.71  ($\text{±}$1.49) | 13.66  ($\text{±}$2.25) |

**S1 Table. Tissue corrected metabolite concentrations for each intervention group** $\text{±}$ **1 standard deviation.** Mean metabolite concentration for GABA and Glx in iu at three experimental time points for each intervention group; anodal tDCS (a-tDCS), anodal high definition tDCS (HD-tDCS) and sham.
